# The Vital Link Between Chronic Disease and Depressive Disorders

**Published:** 2004-12-15

**Authors:** Daniel P Chapman, Geraldine S Perry, Tara W Strine

**Affiliations:** Emerging Investigations and Analytic Methods Branch, Division of Adult and Community Health, National Center for Chronic Disease Prevention and Health Promotion, Centers for Disease Control and Prevention; Division of Adult and Community Health, National Center for Chronic Disease Prevention and Health Promotion, Centers for Disease Control and Prevention, Atlanta, Ga; Division of Adult and Community Health, National Center for Chronic Disease Prevention and Health Promotion, Centers for Disease Control and Prevention, Atlanta, Ga

## Abstract

**Introduction:**

Chronic diseases have assumed an increasingly important role in public health research and intervention. Without treatment, depressive disorders characteristically assume a chronic course and are expected, by 2020, to be second only to heart disease in the global burden of disease. Thus, understanding the relationship between depressive disorders and chronic disease appears vital to public health assessment and health care delivery.

**Methods:**

Articles for review were primarily identified by a Medline search emphasizing the subject headings *mental disorders* or *depression* crossed with selected chronic diseases and conditions including asthma, arthritis, cardiovascular disease, cancer, diabetes, and obesity.

**Results:**

Mental illnesses — most specifically, depressive disorders — were associated with increased prevalence of chronic diseases. This association between depression and chronic disease appears attributable to depressive disorders precipitating chronic disease and to chronic disease exacerbating symptoms of depression. The complex interrelationship between depressive disorders and chronic disease has important implications for both chronic disease management and the treatment of depression.

**Conclusion:**

Depressive disorders assume an important role in the etiology, course, and outcomes associated with chronic disease. Multivariate community-based research and intervention fostering the detection and treatment of depressive disorders is needed, as is further examination of the role exerted by mental illnesses other than depression in the pathogenesis of chronic disease.

## Introduction

Recent research indicates that seven out of 10 office visits to a primary care practitioner concern chronic diseases (1). As the management of chronic diseases has assumed an increasingly vital role in health care delivery, recognition of the importance of depressive disorders has also grown. By 2020, depression is expected to be second only to heart disease as a source of the global burden of disease (2). As chronic disease and depressive disorders are increasingly recognized as major impediments to health, understanding the connection between them becomes of utmost importance to providing quality health care.

Despite the growing recognition of the importance of both chronic disease and depressive disorders to the health of individuals and communities, research examining their interrelationship has been the subject of surprisingly little empirical review. A Medline search for literature reviews emphasizing both chronic disease and depression yielded only two publications. These articles addressed factors germane to health-service costs (3) and individual characteristics precipitating the onset of depressive disorders subsequent to the development of chronic disease (4). While raising important concerns, previous reviews were deliberately limited in scope and did not generally address disease-specific variables potentially underlying the associations between depressive disorders and a number of chronic diseases. To better address this issue, we reviewed the research literature examining the relationships between depressive disorders and prevalent chronic diseases that are also of programmatic relevance to the work of the Centers for Disease Control and Prevention.

## Methods

Articles included in this review were primarily identified through a Medline search of the terms *mental disorders* or *depression* crossed with the chronic diseases and conditions asthma, arthritis, cardiovascular disease, cancer, diabetes, and obesity. These chronic diseases were selected because they have been identified as highly prevalent and as major sources of morbidity and mortality among U.S. adults. Studies included for review were generally limited to empirical investigations that provided definitional or diagnostic criteria for both depression and relevant chronic diseases and that featured a specified time course.

## Results

### Asthma

Nearly 50% of asthma patients may suffer from clinically significant depressive symptoms (5), which have been, in part, attributed to the stress of having a chronic illness (6). In particular, persons with asthma who experience disruptive symptoms, such as dyspnea and nighttime awakening, are at increased risk for major depression (7). The presence of depression among persons with asthma assumes particular gravity because increased depressive symptoms have been associated with poorer asthma outcomes (8), such as impaired voluntary activation of the diaphragm (9). In clinical samples of children and adolescents, asthma has been associated with the presence of an anxiety disorder (10) and with anxious depressive symptoms among youth with moderate and severe persistent asthma (11).

It appears that the symptoms — rather than the diagnosis — of asthma are associated with depression or anxiety (12): 87.5% of persons with frequent asthma attacks manifest psychopathology, compared with 25% of persons with less frequent attacks (13). Similarly, among persons whose asthma is difficult to control, psychopathology — primarily anxiety and depressive symptoms — was associated with more frequent visits to primary care providers and emergency departments and with more hospitalizations (14). Psychological morbidity is associated with poor adherence to medication regimens (15), and mothers of children with asthma are at risk for increased depressive symptoms (16). Early assessment and intervention addressing depressive disorders improves treatment adherence and outcomes and may also decrease mortality (17).

Cognitive behavioral therapy (CBT) — in which the individual is instructed to monitor and challenge self-negating thoughts — has yielded a significant decrease in asthma symptoms and depression (18). Likewise, physical inactivity has been speculated to augment the strength of the association between perceived stressors and depression in persons with asthma, suggesting that exercise may ameliorate this association and decrease the likelihood of depression in this population (19).

### Arthritis

Depression and/or anxiety are among the most commonly reported concerns by persons with arthritis (20). Screening of patients with arthritis revealed that depression was associated with activity restriction, further suggesting that nonpsychiatric physicians should be aware of the mental health status of patients with chronic illnesses (21). Persons with arthritis experiencing arthritis-based disability (22) and the recurrence of arthritic symptoms (23) reported greater depression. Research on adolescents and young adults with arthritis has found that functional status is significantly correlated with depression, self-esteem, and loneliness (24); significantly greater depression was reported among those experiencing more severe symptoms (24).

Research on the Arthritis Self-Management Program found that participation in this intervention had a positive effect on perceptions of self-efficacy, communication with physicians, fatigue, anxiety, pain, and depression (25). A randomized trial of an intervention designed to improve mood (antidepressant medications and/or six to eight sessions of psychotherapy) improved depression and fostered improvements in functional status and quality of life (26). CBT has proven particularly effective in ameliorating depressive symptoms when initiated early in the course of rheumatoid arthritis (27) and when tailored to the concerns reported by persons with rheumatoid arthritis, such as fatigue or mood (28). Similarly, antidepressant medication has been associated with significant improvements in both psychological status and health status in persons with rheumatoid arthritis (29).

Rest and inactivity were previously considered to be reasonable therapeutic approaches in the management of osteoarthritis until it was recognized that physical inactivity contributed to disability and impaired functioning. Subsequent research suggests that a tailored program of aerobic or resistance-based exercise may be an important component of self-managing osteoarthritis (30). Aerobic exercise has been found to both ameliorate depressive symptoms and to reduce disability and pain among persons with arthritis (31).

### Cardiovascular disease

Depressive disorders have been associated with risk factors for cardiovascular disease (CVD), such as smoking and physical inactivity (32), and mental illness, in general, has been associated with increased mortality due to CVD (33). In general, persons who are depressed are much more likely to develop coronary artery disease (34), and meta-analyses reveal that the relative risk for developing heart disease in individuals with depression or depressive symptoms is approximately 1.6 times greater than among nondepressed persons (35,36), which is more than the risk conferred by passive smoking (36). A stronger effect size was reported for clinical depression than for depressive symptoms, suggesting the presence of a dose-response relationship (35). Depression has been positively associated with the metabolic syndrome among women (but not men) younger than 40 years (37), suggesting that early detection and treatment of depression may potentially forestall the risk of cardiovascular disease among women.

Depression or depressive symptoms are also predictive of stroke (38): persons with significant depressive symptoms are approximately twice as likely as those with few depressive symptoms to have a stroke within 10 years (39). Moreover, depression is associated with an increased risk for stroke morbidity and mortality (40).

In addition to being a predictor of stroke, depression commonly develops after a stroke, especially after a stroke affecting the left hemisphere of the brain (41). More than half of patients experiencing a stroke report depressive symptoms within 18 months of having a stroke (42). Post-stroke depression has been associated with impairments in response to rehabilitation (43) and with increased mortality up to two years following the stroke (44). Antidepressant treatment of post-stroke depression is warranted and, in addition to alleviating depression, may foster recovery of cognitive function (45) and significantly increase survival (46).

Depressive disorders also appear related to the occurrence of heart attack, or myocardial infarction (MI). Persons with a history of major depression are more than four times as likely to have an MI than those with no history of depression (47), and high levels of depressive symptoms are associated with an increased risk of MI (48).

Approximately one in six persons who have experienced an MI suffer from major depression, and at least twice that many experience significant depressive symptoms (49). Patients who have had an MI and are also depressed have more medical comorbidities (50) and cardiac complications (51) and are at greater risk for mortality (52) than their nondepressed peers. Increased mortality is also evident in persons who had an MI and who manifest very low levels of depressive symptoms (53), underscoring the importance of mental health to physical health outcomes.

Persons with depression following an MI are less likely to adhere to recommended lifestyle and behavioral changes, potentially increasing their risk for subsequent cardiac events (54). This is particularly unfortunate because cardiac rehabilitation has been found to improve depressive symptoms (55). However, the use of a specific class of antidepressant medications — the selective serotonin reuptake inhibitors (SSRIs) — may, in addition to their beneficial effect on depression, exert antiplatelet effects protecting against MI (56). In addition to being safer in overdose (57), SSRIs are also less likely to induce arrhythmia than other classes of antidepressant medications (58). It has further been concluded that the combination of CBT with an SSRI is frequently the most effective treatment of depression in persons with CVD (59).

### Cancer

Estimates of the prevalence of psychiatric disorders among persons with cancer vary widely, depending on the type of cancer and its clinical stage. Previous research indicates that nearly 50% of patients newly admitted to a cancer center met diagnostic criteria for a psychiatric disorder. Adjustment disorders — distress related to a specific precipitant — comprised 68% of these diagnoses, although many of those diagnosed reported anxiety or depression as a central symptom (60). Among cancer patients judged terminally ill, 53% met psychiatric diagnostic criteria, with delirium — a fluctuating change in cognition and disturbance in consciousness — being the most frequently diagnosed disorder (61).

In addition to delirium, cancer patients also suffer from depression and anxiety (62); 21% of cancer patients are reported to be depressed (63). Depression assumes particular significance in the care of individuals with cancer, because it has been associated with a desire for hastened death among terminally ill cancer patients (64), and increased depressive symptoms are inversely related to survival (65). Of cancer patients in an intensive care unit who were assessed as being at high risk for hospital death, 40% reported depression (66), suggesting that diagnosis and treatment of depression are inadequate. Strikingly, among cancer patients undergoing chemotherapy and experiencing anemia-related fatigue, improved hemoglobin levels have been reported to reduce depressive symptoms (67), further suggesting the importance of physical health to mental health status.

A previous survey of psychotropic prescription practices at five major oncology centers revealed hypnotics to be the most widely prescribed drugs, with antidepressants comprising only 1% of psychotropic prescriptions (68). Subsequent research, however, has indicated an increase of antidepressant use in community cancer care, with 19.2% of breast, 11% of colon, and 13.7% of lung cancer patients receiving antidepressants during a two-year interval (69).

Despite the observation that both antidepressants and psychotherapy are effective in treating depression in patients with cancer, research on antidepressant pharmacotherapy and psychotherapy among persons with cancer has been characterized as largely lacking randomized placebo-controlled trials (70). Moreover, antidepressant prescription has been found to be associated with factors not specifically related to psychopathology, such as patient age or the presence of pain (69), and some speculate that most depressed patients with cancer do not need medication (71). This belief, however, may reflect the misconception that depression is a "natural" response to cancer and does not merit systematic diagnosis and treatment (72).

Research suggests that depression in persons with cancer is amenable to treatment. Among cancer patients with a life expectancy of at least 12 months, CBT has been associated with significantly decreased depressive symptoms across a four-month interval (73). CBT has also been associated with decreased pain, reduced symptomatic distress (74), and subsequent improvement in cellular immune function (75).

Adoption of a depression screening program and antidepressant algorithm by oncologists resulted in significant improvements in mood and quality of life among cancer patients (76). Similarly, a placebo-controlled trial of antidepressant medication in advanced cancer patients demonstrated that antidepressant therapy decreased depressive symptoms and improved patient assessments of quality of life (77). In addition to reducing the risk of depression, data suggest that physical activity may also decrease the risk of colon, breast, and lung cancer (78).

### Diabetes

Elevated rates of depression have consistently been associated with diabetes (79), with results of a meta-analysis indicating depression is twice as prevalent among persons with diabetes than it is among persons without diabetes (80). While it has been proposed that depressive symptoms may be a risk factor for the development of diabetes, this association is most pronounced at high levels of depressive symptoms and, interestingly, only observed among persons with less than a high school education (81). These findings suggest that factors associated with low socioeconomic status may contribute to the development of diabetes among persons with substantial depressive symptoms.

Comorbid depressive symptoms or depression among persons with diabetes have been associated with adaptation to the illness (82), diabetic-related complications (79), unemployment (83), and illness intrusiveness, a construct defined as the degree to which diabetes disrupts valued activities and interests (84). As is true in the general population, depression was more prevalent among women than among men with diabetes (80,85) and among younger adults (85). Depressive symptoms are more likely to persist among persons with multiple diabetic-related complications and those with less than a high school education (79). In a prospective community-based study, baseline depressive symptoms were positively associated with fasting insulin levels and physical inactivity (86). A diagnosis of diabetes and self-reported depression were positively associated with sedentariness in both bivariate and multivariate analyses (87). Compared with their nondepressed peers, patients with diabetes who were diagnosed with depression were more likely to report frequent overeating of sweets and high-fat foods and were less satisfied with their ability to adhere to a diabetic diet away from home (88).

Despite the availability of measures to screen for depression, it is estimated that less than 25% of those with depression are diagnosed and treated (89). This is particularly disconcerting because the treatment of depression appears to be associated with improved glycemic control (90). Furthermore, because depression is associated with diabetic complications (91), treatment of depression may also reduce diabetes-related disability. Compared with their nondepressed peers, persons with diabetes and depression have higher ambulatory-care use and fill more prescriptions. Total health expenditures for persons with diabetes and depression were 4.5 times higher than for those without depression: $247 million compared with $55 million (85).

Research has revealed that both CBT (90) and antidepressant pharmacotherapy (92) are associated with decreased severity of depression among persons with diabetes and with improved glycemic control. Thus, in addition to preventing needless suffering, the treatment of depression among persons with diabetes offers the added promises of substantial financial savings and improved medical care of these individuals.

### Obesity

Several studies have indicated an association between psychopathology, including depressive symptoms, and high body mass index (BMI), or obesity. The relationship between obesity and psychopathology differs among men and women, with a BMI ≥30 among women associated with nearly a 50% increase in the lifetime prevalence of depressive disorders compared with nonobese women (93). In contrast, while BMI has not been found to be related to measures of mental well-being among men, abdominal obesity or a high waist/hip ratio has been associated with an increased prevalence of both depressive symptoms (94) and antidepressant medication use (95).

Although it is important to note that most overweight or obese persons do not suffer from mood disorders (96), significant positive associations have been reported between BMI and depressive symptoms (97). It has been posited that a common pathophysiology may underlie both obesity and depression. The neurotransmitters serotonin and norepinephrine are involved in regulating both mood and body weight and, logically, in the treatment of both depression and obesity (98). Antidepressant medications available before the development of SSRIs frequently induced weight gain; newer agents generally do not stimulate appetite, thus making them potentially useful in depressed patients who do not wish to gain weight (99).

Previous longitudinal research has examined the relationship between depressive symptoms or psychological well-being and weight gain. Women who were either normal weight or overweight at baseline and who had experienced a recent weight gain scored lower on a measure of psychological well-being than women who had not gained weight (100). Similarly, persons who were overweight and depressed at baseline demonstrated a significantly increased likelihood of subsequent weight gain relative to those who were not depressed. Among the highest quintile of baseline BMI, this relationship was stronger among women (with an odds ratio of 2.2) than men (with an odds ratio of 1.3) (101).

Cognitive behavioral interventions have been useful in managing obesity, largely by modifying eating behaviors and dietary choices in addition to decreasing psychological distress and sedentariness (102). In addition to fostering weight loss, CBT has been found to improve self-reported mental health among obese persons (103). However, psychosocial difficulties have been associated with weight gain following initial weight loss among obese individuals who had received CBT (104), with long-term CBT compliance being particularly low among persons with binge-eating behaviors (105).

Strikingly, children and adolescents with major depressive disorder appear to manifest an increased risk for subsequently becoming overweight (96), suggesting that both depressive disorders and their treatment are relevant to the prevalence of obesity. The relationship between obesity and depressive disorders thus appears to be reciprocal, with advances in the recognition and treatment of each of these diseases potentially fostering improved mental and physical health.

## Discussion

Research examining the association between depressive disorders and chronic disease suggests that timely diagnosis and treatment of psychiatric disorders could greatly affect the impact of chronic disease. The presence of mental illness may be an important contributor to the etiology of chronic disease. Thus, the promotion of mental health would likely result in reducing a considerable proportion of the burden of chronic disease. Similarly, the presence of depressive disorders often adversely affects the course and complicates the treatment of chronic disease. It is important to remember that untreated depressive disorders characteristically assume a chronic course (106), thereby adding to the burden of chronic disease in their own right. Multivariate investigation of the associations among depressive disorders, chronic disease, and a variety of medical and sociodemographic characteristics would provide valuable insights into contemporary notions of health and quality of life.
